# Role of dopamine in the pathophysiology of Parkinson’s disease

**DOI:** 10.1186/s40035-023-00378-6

**Published:** 2023-09-18

**Authors:** Zhi Dong Zhou, Ling Xiao Yi, Dennis Qing Wang, Tit Meng Lim, Eng King Tan

**Affiliations:** 1 National Neuroscience Institute of Singapore, 11 Jalan Tan Tock Seng, Singapore, 308433 Singapore; 2 Department of Neurology, Singapore General Hospital, Outram Road, Singapore, 169608 Singapore; 3 Signature Research Program in Neuroscience and Behavioral Disorders, Duke-NUS Graduate Medical School Singapore, 8 College Road, Singapore, 169857 Singapore; 4 Department of Neurology, Zhujiang Hospital, Southern Medical University, Guangzhou, 510280 China; 5 Department of Biological Science, National University of Singapore, Singapore, 119077 Singapore

**Keywords:** Dopamine, Dopamine quinones, 3,4-Dihydroxyphenylacetaldehyde, Neurodegeneration, Pathogenesis, Parkinson's disease, Reactive oxygen species, Therapeutic strategies

## Abstract

A pathological feature of Parkinson’s disease (PD) is the progressive loss of dopaminergic neurons and decreased dopamine (DA) content in the substantia nigra pars compacta in PD brains. DA is the neurotransmitter of dopaminergic neurons. Accumulating evidence suggests that DA interacts with environmental and genetic factors to contribute to PD pathophysiology. Disturbances of DA synthesis, storage, transportation and metabolism have been shown to promote neurodegeneration of dopaminergic neurons in various PD models. DA is unstable and can undergo oxidation and metabolism to produce multiple reactive and toxic by-products, including reactive oxygen species, DA quinones, and 3,4-dihydroxyphenylacetaldehyde. Here we summarize and highlight recent discoveries on DA-linked pathophysiologic pathways, and discuss the potential protective and therapeutic strategies to mitigate the complications associated with DA.

## Introduction

Parkinson’s disease (PD) is a common neurodegenerative movement disorder that affects 1% of the population above 60 years. PD is characterized by the progressive loss of dopaminergic neurons and the formation of Lewy bodies in the affected brain areas [1, 2]. Dopamine (DA), a brain hormone with a chemical formula C_8_H_11_NO_2_, is synthesized by substantia nigra (SN) dopaminergic neurons which have axon projections in the striatum [3]. As a brain neurotransmitter, DA is released from the presynaptic membrane to the synaptic cleft, where it binds and activates DA receptors on the postsynaptic membrane [3]. Progressive degeneration of dopaminergic neurons reduces DA content in the SN and striatum and triggers the onset of PD clinical symptoms such as tremor, postural instability, bradykinesia and muscle rigidity [4]. PD is an incurable neurodegenerative disease and the levodopa (*L*-DOPA) therapy can only alleviate PD symptoms, without any therapeutic improvements on the progression of DA neuronal degeneration [5].

The exact PD pathogenesis remains to be clarified. However, evidence shows that oxidation of endogenous DA can induce specific oxidative stress in dopaminergic neurons [6–8]. DA oxidation can occur spontaneously or be mediated by enzymes or metal ions, producing deleterious DA oxidative by-products [5, 9]. Many reactive DA metabolites are toxic to dopaminergic neurons, including reactive oxygen species (ROS), DA quinones (DAQs) and 3,4-dihydroxyphenylacetaldehyde (DOPAL) [5, 9]. ROS can induce oxidative stress, whereby highly reactive DAQs and DOPAL can covalently conjugate with cysteine, lysine and tyrosine residues of proteins, leading to misfolding, cross-linking, functional inactivation and aggregation of affected proteins [10–12]. DA impairs the functions of mitochondria, ubiquitin–proteasome system (UPS), lysosome and autophagy, resulting in DA neuron vulnerability [10–12]. DA and its derivatives are involved in the toxity of PD-related neurotoxins, such as rotenone, 1-methyl-4-phenyl-1,2,3,6-tetrahydropyridine (MPTP) and iron species [13, 14]. DA is also involved in the PD pathogenesis associated with genetic factors, including *SNCA* (encoding α-synuclein [α-syn]), *LRRK2* (leucine-rich repeat kinase 2), *PINK1* (PTEN-induced kinase 1), *Parkin*, *DJ-1* and *GBA1* (glucocerebrosidase-1 [GCase]), contributing to DA neuronal degeneration [15–19]. In this review, we briefly summarize the role of DA metabolic pathways in PD pathophysiology and discuss therapeutic strategies to protect dopaminergic neurons and mitigate the complications associated with DA synthesis, transportation, storage and metabolisms.

## DA metabolic pathways

DA is the neurotransmitter for signal transduction in dopaminergic neurons. It is formed as an intermediate during the formation of norepinephrine and epinephrine [20]. DA is synthesized by two steps in catecholamine neurons. First, the amino acid tyrosine is converted to *L*-DOPA by tyrosine hydroxylase (TH). Subsequently, *L*-DOPA is decarboxylated to DA by aromatic amino acid decarboxylase [21]. In the resting state, the synthesized DA is transported into and stored in vesicles by vesicular monoamine transporter 2 (VMAT2) in the cytosol of dopaminergic neurons, facilitated by a vesicular ATPase-dependent H^+^ gradient. VMAT2, when present on synaptic vesicles, acts as a stoichiometric antiporter under acidic circumstances, transporting two H^+^ ions out and one monoamine molecule into vesicles [22]. Deficiency of VMAT2 elevates monoamine turnover, evidenced by reduced levels of DA, norepinephrine, 5-hydroxytryptamine and histamine in catecholaminergic neurons, leading to up-regulation of amine-synthesizing enzymes [23]. Under neuronal activation, DA is released into the synaptic cleft for signal transduction. The released DA can be taken up and degraded by neighboring astrocytes and microglia or absorbed back into the vesicles of the presynaptic neurons via DA transporters (DATs) for re-use. DA in the cytosol is unstable and will undergo oxidation [24].

DA can undergo auto-oxidation, especially under basic condition, to generate small-molecule ROS and highly reactive DAQs [6]. DA oxidation can be facilitated by enzymatic catalysis (such as tyrosinase) or mediated by transition metal ions (iron, copper and manganese ions) [5]. Briefly, the oxidation of DA initiates from the desquamation of two protons and electrons from the hydroxyl groups of DA to form DA-*o*-quinone (DOQ), a highly reactive, undetectable species with a very short lifespan, and generates ROS [5]. DOQ can be reversed back to DA by ambient reductants or further oxidized to form reactive aminochrome (AM), a kind of cyclized DAQ, via internal cyclization of DOQ under insufficient ambient reductive forces [5]. AM is more stable than DOQ and can be detected, monitored and characterized. DOQ and AM can react and conjugate with many biomolecules, including protein residues cysteine and tyrosine with sulfhydryl and hydroxyl groups, whereas polymerization of AM will form neuromelanin (NM), an insoluble granular pigment in SN [5, 25, 26].

NM has been reported to prevent the neurotoxicity of DAQs and is considered as an anti-oxidative agent since it directly binds and inactivates radical species under normal conditions [27]. Interestingly, NM also generates free radical species under oxidative stress conditions [28]. Another study suggests that NM may be involved in α-syn-associated DA neuronal damage [29]. In NM-producing rats that show PD pathology when NM accumulates above a specific pathogenic threshold, the time-dependent accumulation of NM and degeneration of dopaminergic neurons under overexpression of tyrosinase, were significantly alleviated by viral vector-mediated overexpression of VMAT2 [30]. Furthermore, the reduced NM generation was associated with decreased formation of Lewy body-like inclusions and improved survival of dopaminergic neurons and motor functions in rats [30]. These findings highlight the potential pathological roles of NM accumulation in PD, suggesting therapeutic potentials of inhibiting time-dependent NM accumulation for PD.

**Fig. 1:**
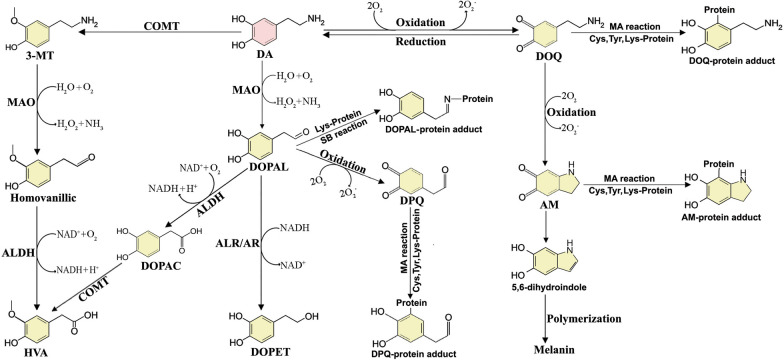
DA metabolic pathways. DA is unstable and can undergo oxidation to produce reactive DOQ and ROS. The reaction can be reversed under sufficient ambient reductive force. The reactive DOQ can covalently conjugate with protein residues via MA reaction to form DOQ-protein adducts. DOQ can further be oxidized to generate ROS and AM, a cyclized DAQ. AM can also conjugate with protein residues to form AM-protein adducts via MA reaction or undergo internal rearrangement to form 5,6-dihydroxyindole, which can further polymerize to form melanin. Alternatively, DA can be catalyzed by COMT to form non-toxic 3-MT and finally HVA. DA can also be catalyzed by MAO to form reactive DOPAL and ROS. DOPAL can be catalyzed by ALDH to form non-toxic DOPAC and generate HVA under the catalysis of COMT. DOPAL can also be reduced by ALR/AR to form inactivate DOPAE. However, DOPAL is reactive and can conjugate with protein lysine residues to form DOPAL-protein adducts via SB reaction. DOPAL can be further oxidized to generate reactive ROS and DPQ, while DPQ can conjugate with protein residues via MA reaction to form DPQ-protein adducts. *ALDH*  aldehyde dehydrogenase, *AM* aminochrome, *COMT* catechol-*o*-methyltransferase, *DA *dopamine, *DOPAL* 3,4-dihydroxyphenylacetaldehyde, *DOQ* DA-*o*-quinone, *DPQ* DOPAL-quinone, *HVA* homovanillic acid, *3-MT* 3-methoxytyramine

Besides, the cytosol DA is catalyzed by monoamine oxidases (MAO) to produce DOPAL, a reactive and toxic DA metabolite, and ROS [9]. DOPAL can be monitored, with physiological concentrations around 2–3 μM in dopaminergic neurons in the SN. DOPAL concentrations higher than 6 μM are toxic to many cell lines [31]. As a quite electrophilic molecule, DOPAL can conjugate with lysine and cysteine residues to induce toxicity. However, DOPAL can be detoxified by NAD(P)^+^-dependent aldehyde dehydrogenase (ALDH) or reduced by aldehyde/aldose reductase (ALR/AR) to form the inactive 3,4-dihydroxyphenylethanol, or further oxidized to non-toxic 3,4-dihydroxyphenylacetic acid where the aldehyde moiety is replaced by a carboxyl group [32]. Alternatively, DA released into the synaptic cleft can be absorbed into the surrounding glial cells, in which DA can be catalyzed by catechol-*o*-methyltransferase and MAO to form non-toxic 3-methoxytyramine and finally homovanillic acid [5, 33]. The detailed DA metabolic pathways are illustrated in Fig. 1.

## The pathogenic roles of DA in PD

DA-relevant toxicity in PD pathogenesis has been demonstrated in multiple in vitro and in vivo PD models [34, 35]. Direct injection of DA (up to 1 μmol) into the rat striatum led to the loss of dopaminergic nerve terminals and the formation of cysteinyl adducts of DA in a DA-dose-dependent manner, and this was alleviated by co-administration of glutathione (GSH) or ascorbate [36–38]. Injection of AM, a cyclized DAQ, into the SN of rats induced dopaminergic neuronal degeneration and motor impairment [39, 40]. Injection of DOPAL into the rat SN region resulted in DA neuronal loss [41]. Furthermore, deregulation of endogenous DA synthesis, storage, transportation and metabolism by pharmacological and genetic approaches can lead to deleterious effects on dopaminergic neurons. Both in vitro and in vivo studies showed that overexpression of TH, a rate-limiting enzyme in dopamine biosynthesis, induces degeneration of dopaminergic neurons [42, 43]. The Tet-on-induced TH overexpression in human midbrain-like organoids derived from the induced pluripotent stem cells (iPSCs) has also been shown to induce degeneration of dopaminergic neurons [44]. Consistently, RNAi knockdown of TH significantly alleviates the rotenone- and mutant α-syn-induced degeneration of dopaminergic neurons in a *Drosophila* PD model [35]. Knockdown of VMAT2 in mice disturbs DA vesicle storage and leads to mild and progressive DA neurodegeneration, accompanied by elevated levels of cysteinyl-DAQs adducts, suggesting enhanced DA oxidation and DAQ toxicity [45]. Furthermore, VMAT2-knockout mice are sensitive to the neurotoxic drug methamphetamine [46, 47], which promotes DA redistribution from synaptic storage vesicles into the cytosol [48, 49]. However, VMAT2 overexpression protects against the methamphetamine-induced toxicity to dopaminergic neurons [50]. The degeneration of dopaminergic neurons can be induced by overexpression of DATs, which enhance DA re-uptake to increase cytosolic DA levels in PD models in vivo [51, 52]. Furthermore, disturbances of the ALDH-catalyzed detoxification of DOPAL, a reactive DA metabolite, can be pathogenic. Mice lacking both cytosolic and mitochondrial Aldh, two key enzymes to detoxify DOPAL in the brain, exhibit degeneration of dopaminergic neurons in the SN and development of age-dependent parkinsonian phenotypes [53]. A clinical study involving 360 PD patients and 754 normal controls demonstrated a positive association between environmental exposure to benomyl, a potent ALDH inhibitor, and the increased risk of PD [54].

DA exerts toxicity via its deleterious metabolic by-products, including reactive ROS, DAQs and DOPAL. ROS generated from DA oxidation can aggravate oxidative stress, which has been evidenced by postmortem studies reporting that oxidative modifications have significant and comprehensive impacts on nucleic acids, lipids, proteins and GSH in PD patient brains [55]. However, DAQs, rather than small-molecule ROS, are more significant pathological factors in degeneration of dopaminergic neurons [12]. The DA-derived DAQs can irreversibly conjugate to the sulfhydryl groups of cysteine residues via Michael-addition (MA) reaction, leading to protein misfolding and loss of function [56, 57]. Our recent findings suggest, for the first time, that DAQs can also conjugate with hydroxyl groups of tyrosine and serine, especially hydroxyl groups in the phenol ring of tyrosine [26]. In the study, we synthesized three similar peptide fragments with 30 amino acids without *L*-cysteine but containing five serine residues (peptide S, HGKKQDNRSQESGEDGDDREGSGKSNESQD), five tyrosine residues (peptide Y, HGKKQDNRYQEYGEDGDDREGYGKYNEYQD) or glycine residues (peptide G, HGKKQDNRGQEGGEDGDDREGGGKGNEGQD) [26]. The peptides were incubated with DA in the presence or absence of tyrosinase. After incubation, the conjugation of DAQ to the peptides was  monitored by nitroblue tetrazolium staining plus polyacrylamide gel electrophoresis analysis [26]. We found that the peptide G with glycine residues could not react with DAQs, while the peptide S with five serine residues had weak capabilities to conjugate with DAQs [26]. However, the peptide Y with five tyrosine residues could effectively conjugate with DAQs [26]. These results provide direct evidence that DAQs can conjugate with the hydroxyl groups of protein residues, especially the tyrosine residue with a hydroxyl group in a phenol ring. Furthermore, the hydroxyl groups from tea polyphenols can competitively conjugate with DAQs to protect against DAQ-induced protein modifications, further supporting the reactions between DAQs and phenolic hydroxyl groups of protein residues [26]. The peptide G has three lysine residues; however, no conjugations of DAQs were identified. Therefore, Schiff-base (SB) reactions between DAQs and peptides can be excluded. The conjugations between DAQs and tyrosine residues occur via the MA reaction, similar to reactions between DAQs and cysteine.

The DAQ-modified proteins are involved in the DA-induced toxicity to human dopaminergic neurons [58]. DAQs can conjugate with αB-crystallin and heat shock protein 27 (HSP27), two small heat-shock chaperone proteins, to promote the cross-linking of αB-crystallin and HSP27 and inhibit their chaperone functions [59]. Moreover, DAQs can inhibit mitochondrial, lysosomal, autophagy and UPS functions in dopaminergic neurons [12, 60–62]. DAQs can irreversibly inhibit the activities of proteasomes, whereas small-molecule ROS only induce reversible proteasome inhibition [11]. In a previous study using SH-SY5Y cells and isolated mouse liver and rat brain mitochondria, DAQ treatment altered the morphology of mitochondria, induced mitochondrial membrane depolarization and opening of the mitochondrial transition pore (MTP), and inhibited mitochondrial ATP synthesis [63]. In another recent study, DA oxidation was identified to mediate the mitochondrial and lysosomal dysfunction in PD patients [10]. The enhanced mitochondrial oxidative stress leads to DA oxidation with generation of DAQs, conjugation of DAQs with GBA1, inhibition of GBA1 enzymatic activity, lysosomal dysfunction, and accumulation of deleterious α-syn protein [10]. DAQs can inhibit autophagy via reactions with α- and β-tubulin, as α- and β-tubulin mediate microtubule aggregation for the fusion of autophagy vacuoles with lysosomes [62]. Furthermore, DAQs can directly conjugate with α-syn to induce the formation of toxic α-syn protofibrils, leading to inactivation of the chaperone-mediated autophagy [62].

**Fig. 2:**
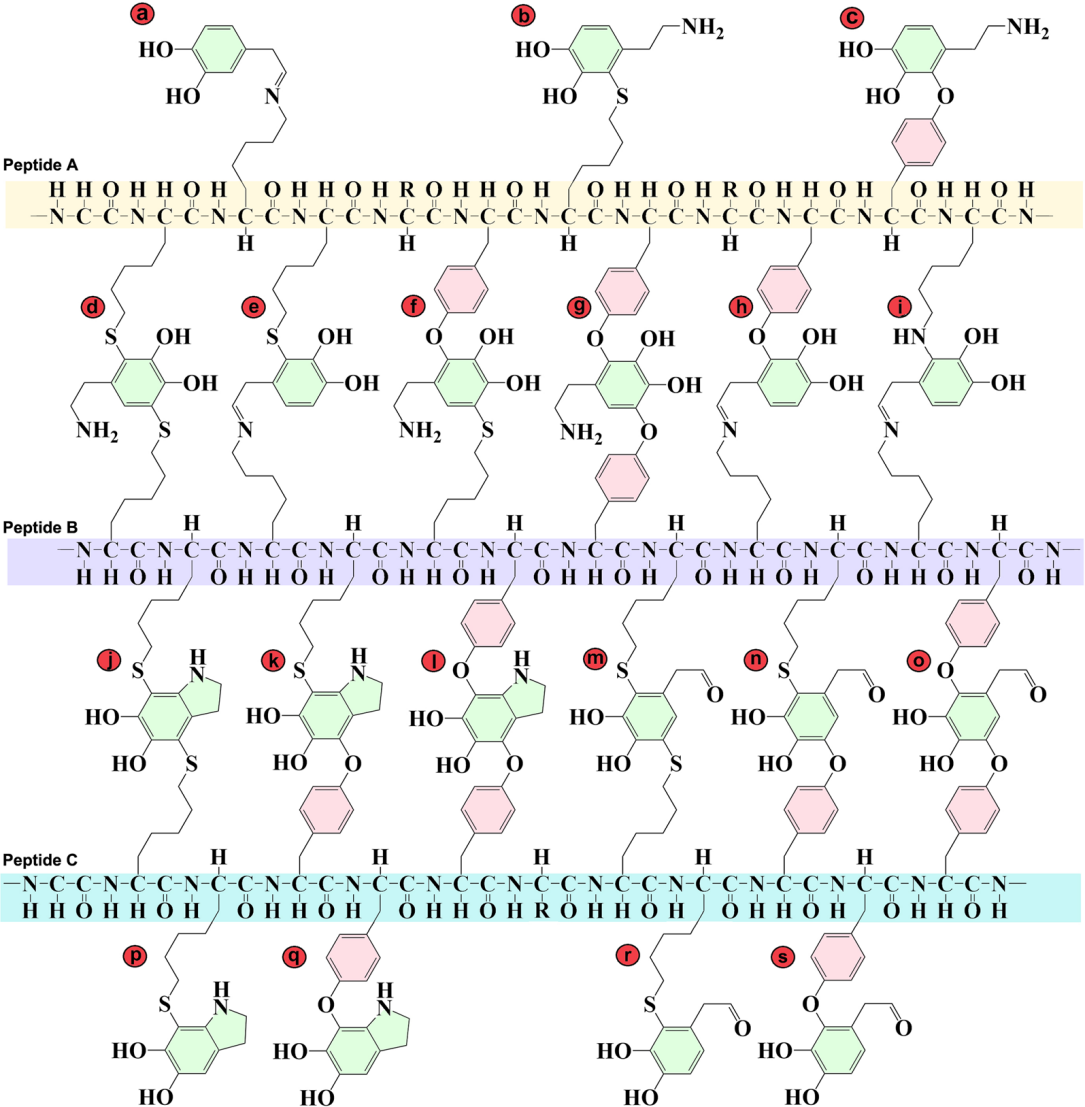
Hypothetical conjugations of DAQs and DOPAL with protein residues, leading to protein modifications and cross-linking. **a**–**c** DAQ or DOPAL conjugations to peptide A. **a** SB adductive reaction of DOPAL with lysine; **b** MA adductive reaction of DOQ with cysteine; **c** MA adductive reaction of DOQ with tyrosine residue. **d**–**i** DAQ or DOPAL conjugation and cross-linking between peptides A and B. **d** MA adductive reactions of DOQ with two cysteine residues; **e** MA and SB adductive reactions of DPQ with cysteine and lysine residues, respectively; **f** MA adductive reactions of DOQ with tyrosine and cysteine residues; **g** MA adductive reactions of DOQ with two tyrosine residues; **h** MA and SB adductive reactions of DPQ with tyrosine and lysine residues, respectively; **i** MA and SB adductive reactions of DPQ with two lysine residues. **j**–**o** DAQ or DOPAL conjugations and cross-linking between peptides B and C. **j** MA adductive reactions of AM with two cysteine residues; **k** MA adductive reactions of AM with cysteine and tyrosine residues; **l** MA adductive reactions of AM with two tyrosine residues; **m** MA adductive reactions of DPQ with two cysteine residues; **n** MA adductive reactions of DPQ with cysteine and tyrosine residues; **o** MA adductive reactions of DPQ with two tyrosine residues; **p**–**s** Conjugations to peptide C. **p** MA adductive reaction of AM with cysteine residue; **q** MA adductive reaction of AM with tyrosine residue; **r** MA adductive reaction of DPQ with cysteine residue;** s** MA adductive reaction of DPQ with tyrosine residue

DOPAL is also reactive and can be an endogenous neurotoxin due to the presence of its both aldehyde and catechol moieties [9]. The neurotoxicity of DOPAL has been reported in various studies both in vitro and in vivo [31, 41]. DOPAL can conjugate through its aldehyde moiety with lysine residues of proteins via the SB reaction [64]. Furthermore, the oxidation of the catechol group of DOPAL can further generate DOPAL-quinone (DPQ), which can conjugate with the sulfhydryl group of cysteine residues via MA reaction [65]. In principle, DPQ, as one of DAQs, should be able to react with the hydroxyl group of tyrosine via MA reaction. DOPAL is involved in multiple mechanisms of DA neurotoxicity [9]. The high reactivity of aldehyde and catechol moieties of DOPAL results in protein cross-linking and aggregation, aggravated proteostatic stress and neurodegeneration [9, 66]. DOPAL has been shown to conjugate with multiple proteins (Table 1) [64, 67], including α-syn. DOPAL conjugates with lysine residues of α-syn forming SB and MA adducts, leading to the formation of α-syn oligomers, aggravating α-syn toxicity, and this can be abrogated by a lysine-blocking strategy [68]. The DOPAL-modified α-syn protein can accumulate in the endo-lysosomal pathway, leading to impaired proteostasis and neurodegeneration of dopaminergic neurons both in vitro and in vivo [69]. The lysine residues undergo many post-translational modifications, including ubiquitination, SUMOylation and acetylation, which are vital for cell survival and proliferation [70]. The conjugation of DOPAL to lysine residues interferes with many cellular events and down-regulates cell viability, as evidenced by ubiquitin oligomerization, accumulation of ubiquitinated proteins and impairment of UPS functions upon DOPAL challenges [71]. Besides, the DOPAL-induced oxidative stress enhancement, enzymatic inhibition, mitochondrial impairment and disturbances of cellular proliferation and death signaling pathways, cooperatively contribute to the DA-relevant toxicity in PD pathogenesis [9]. The complicated conjugations of DAQs and DOPAL with cysteine, tyrosine and lysine residues that result in protein modifications and cross-linking are illustrated in Fig. 2. Proteins modified by DAQs and DOPAL are summarized in Table 1.

**Table 1:** Proteins modified by DAQs and DOPAL

Proteins or peptides	Modified residues	DA oxidation products	References
Apoferritin protein	–	DA	[72]
α-Syn	Lysine	DOPAL	[68, 69, 73–75]
α-Syn	–	DA	[76, 77]
α-Syn	–	DPQ	[78]
α-Syn	Lysine	DPQ	[71]
α-Syn	–	DAQs	[79, 80]
GCase	Cysteine	DAQs	[81]
Parkin	Cysteine	DA	[82]
DJ-1	Cysteine106 and Cysteine53	DAQs	[83]
DJ-1	Cysteine	DAQs	[57, 84]
Chaperonin	Cysteine	DAQs	[57]
Ubiquinol cytochrome *c* reductase core protein 1	Cysteine	DAQs	[57]
Mortalin	Cysteine	DAQs	[57]
Mitofilin	Cysteine	DAQs	[57]
Mitochondrial creatine kinase	Cysteine	DAQs	[57]
Ubiquitin carboxy-terminal hydrolase L1	Cysteine	DAQs	[57]
Glutathione peroxidase 4	–	DAQs	[85]
Actin	–	DAQs	[86]
α-, β-tubulin	–	DAQs	[86]
BSA	Cysteine	DAQs	[87, 88]
BSA	–	DOPAL	[67]
GAPDH	Cysteine	DOPAL	[64, 89]
HSP27	Lysine	DAQs	[59]
αB-crystallin	Lysine	DAQs	[59]
β-lactoglobulin	Cysteine	DAQs	[87]
Synthesized peptide	Tyrosine/Serine	DAQs	[26]
Myoglobin	Cysteine/Histidine	DAQs	[90]
*L*-lactate dehydrogenase	–	DAQs	[91]
Malate dehydrogenase	–	DAQs	[91]
Superoxide dismutase 2	Cysteine	DAQs	[92]
TH	Cysteine	DAQs	[93, 94]
TH	–	DOPAL	[95]
VMAT2	–	DPQ	[71]
GBA	–	DPQ	[71]
Ubiquitin	–	–	[71]
*L*-aromatic-amino-acid decarboxylase	–	DPQ	[71]

Mitochondrial dysfunction, including systemic deficiency of the electron transport chain complex I, is a well-established player in the pathogenesis of both sporadic and familial PD [96–98]. The mitochondrial toxins rotenone and 1-methy-4-phenyl-1,2,3,6-tetrahydropyridine (MPTP), two PD-associated inhibitors of mitochondrial complex I, can induce PD-like phenotypes both in vitro and in vivo [13]. MPTP can cross the blood–brain barrier and be taken up by glial cells, where it is converted to 1-methyl-4-phenylpyridinium (MPP^+^) by MAO-B [99]. MPP^+^ can bind the mitochondrial complex I, leading to ROS generation and mitochondrial dysfunction [100]. However, increased DA content has been identified to enhance the rotenone- and MPP^+^-induced toxicity in various in vitro studies [101, 102]. The oxidation of DA aggravates the dopaminergic cell death induced by MPP^+^ challenge [103], while DA depletion can remarkably alleviate dopaminergic neuronal degeneration caused by mitochondrial complex I inhibitors [104]. Also, downregulation of TH expression by RNAi approach can significantly alleviate the rotenone-induced dopaminergic neuronal degeneration in *Drosophila* [35].

Numerous studies have indicated the vital pathogenic roles of iron species in PD pathogenesis [14]. Accumulation of iron species has been found in the SN region in both post-mortem brain tissues and in living PD patients [105, 106]. Recent evidence demonstrates that iron species can induce DA-relevant toxicity in dopaminergic neurons [107]. DA has been found to promote cellular iron accumulation and enhance oxidative stress responses in macrophages [108]. Iron, as a co-factor of TH, increases TH activity and upregulates DA levels, while this is not seen with other divalent metal ions [109, 110]. Furthermore, iron species, especially free iron ions, form specific iron-DA complexes and subsequently mediate extensive DA oxidation to produce deleterious DAQs and ROS, leading to proteasome inhibition, dopaminergic neuron vulnerability and degeneration [107]. The iron-DA complex formation can be disrupted by iron chelators, thus abrogating the iron species-mediated DA oxidation; however, DAQ-scavenging agents, including GSH and ascorbate, do not have these effects [107]. In contrast, they can alleviate the toxicity of by-products of iron-mediated DA oxidation, suggesting different protective mechanisms and profiles of these neuroprotective agents [107].

Currently, *L*-DOPA replacement is an effective therapeutic strategy to alleviate PD symptoms via enhancing DA levels in PD brains. Whether* L*-DOPA protects or impairs dopaminergic neurons is still under debate. The protective effects of *L*-DOPA have been reported in some PD models [111, 112]. However, some studies also showed toxicity of *L*-DOPA to neurons and non-neuronal cells, as *L*-DOPA can undergo auto-oxidation to generate toxic and reactive ROS and DAQs [113, 114]. In a systems-level computational model of SN-striatum, *L*-DOPA treatment was observed to result in a loss of dopaminergic neuronal terminals in the SN under energy deficiency, which was alleviated by co-administration of GSH [115]. Recently, Hörmann et al. reported that *L*-DOPA can mediate both a neurotoxic and a neuroprotective activity, depending on the oxygen tension. They found that at physiological oxygen levels (which are very distinct to normoxic conditions in all in vitro experiments), *L*-DOPA inhibited mitochondrial functions, suppressed oxidative phosphorylation and depleted the NADH pool, in the absence of *L*-DOPA auto-oxidation and oxidative cell damage [116]. Furthermore, a previous study showed that as a close structural analogue of *L*-tyrosine, *L*-DOPA can be incorporated into synthesized proteins, leading to protein misfolding and inactivation in SH-SY5Y neuroblastoma cells [117]. These findings suggest that the mechanisms of *L*-DOPA toxicity are complicated and warrant further investigations.

## Crosstalk between DA and PD genes in PD pathogenesis

Although most PD cases are sporadic, familial forms of PD caused by genetic mutations account for about 5%–10% of PD cases. So far, multiple PD-associated genes have been identified, including *SNCA*, *LRRK2*, *PINK1*, *Parkin*, *DJ-1* and *GBA1*. Recent studies have suggested interactions between DA and PD-linked genetic factors, which promote neurodegeneration of dopaminergic neurons.

### *SNCA*

The *SNCA* gene encoding α-syn is the first identified PD-related gene. Mutations in *SNCA*, including missense and multiplication mutations, can cause early-onset autosomal-dominant PD [15]. The α-syn protein can form aggregates, which are the major component of Lewy bodies in PD patient brains [118, 119]. Studies have shown that the toxicity of α-syn is DA-relevant [120–122]. Elevated DA levels can aggravate degeneration of dopaminergic neurons induced by either wild-type (WT) or mutant α-syn [121]. A previous study demonstrated that WT α-syn is beneficial to dopaminergic neurons, whereas overexpression of WT α-syn in the presence of DA induce dopaminergic neuronal damage [120, 122]. Inhibition of TH by α-methyltyrosine (α-MT) alleviates WT or mutant α-syn-induced dopaminergic neuron toxicity [120]. DA-derived products have been shown to conjugate with α-syn proteins and stabilize toxic α-syn oligomers, which have been validated in numerous in vitro and in vivo studies [123, 124]. The α-syn protofibrils can form pore-like assemblies on the membranes of intracellular vesicles to impair vesicle integrity, leading to leakage of vesicle contents and dopaminergic neuron vulnerability [125]. DA-derived DAQs and DOPAL can conjugate with α-syn to form unstructured adducts, slowing the conversion of α-syn protofibrils to fibrils and elevating the toxicity of α-syn protofibrils [68, 126]. DOPAL reacts with α-syn protein, leading to α-syn accumulation, proteostasis disturbance and degeneration of dopaminergic neurons in PD models [69]. The increased, DOPAL-modified α-syn has been detected in post-mortem striatal tissues from idiopathic PD patients, correlating with the DA-dependent α-syn pathology [69]. Furthermore, increased DA level has been identified in mice expressing human A53T mutant α-syn [124]. The elevated DA level is positively associated with the formation and toxicity of α-syn oligomers, suggesting a potential adverse DA–α-syn feedback loop in dopaminergic neurons under *SNCA* mutations [124].

### *LRRK* and *PINK1*

LRRK2 and PINK1 are two serine/threonine-protein kinases related to PD. LRRK2 is a widely expressed, large, single-polypeptide protein with multiple domains including ankyrin, leucine-rich repeat, WD40 repeats, and the catalytic core, Ras-of-complex proteins (ROC)-GTPase domain with serine/threonine kinase activities [127]. LRRK2 plays multiple roles in various signaling pathways via phosphorylation of its substrates [128, 129]. The toxicity of LRRK2 mutants is dependent on the increased kinase activity as shown in in vitro and in vivo studies [19]. The dominant G2019S *LRRK2* mutation with increased serine/threonine kinase activity is well-known as the most prevalent cause of genetic factor-induced late-onset sporadic and familial forms of PD [130]. PINK1 is a 68-kDa serine-threonine kinase containing 581 amino acids. *PINK1* mutations can contribute to autosomal recessive, early-onset PD [131]. PINK1 has an N-terminal mitochondria-targeting fragment, followed by a transmembrane domain, a serine/threonine kinase domain and a regulatory C-terminal domain [42]. Most PD-linked *PINK1* mutations are located within the kinase domain, indicating that the PINK1 kinase activity is the key to its neuroprotective roles in dopaminergic neurons [16].

**Fig. 3:**
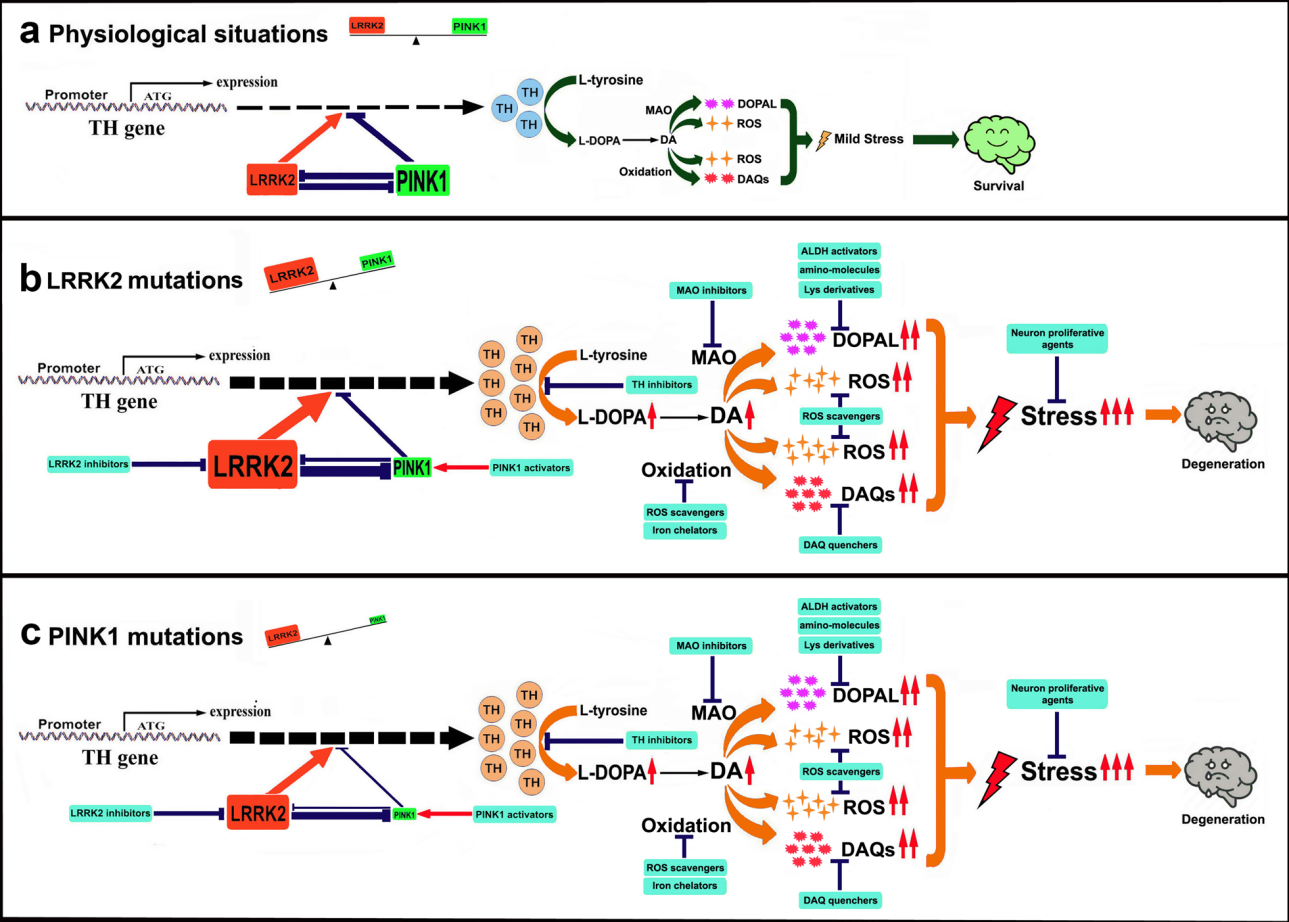
LRRK2-PINK1 kinase pair balance in modulation of TH-DA pathway, significant to PD pathogenesis and therapy. *LRRK2* and *PINK1* form a functional kinase pair balance to regulate TH expression and DA production in DA neurons. **a** *LRRK2* enhances TH expression and DA synthesis, while PINK1 inhibits TH expression and DA production. *LRRK2* and *PINK1* can promote proteasome degradation of each other, leading to a function l kinase balance in steady state. Under physiological conditions, the moderate DA level in brains will cause mild stress to DA neurons by reactive ROS, DAQs and DOPAL generated from DA oxidation and MAO catalyzed DA metabolism. DA neurons can survive and be healthy. **b** However when *LRRK2* is mutated, *LRRK2* kinase activity can be increased to up-regulate TH and DA levels. The increased *LRRK2* kinase activity will also promote *PINK1* degradation to inhibit *PINK1* function. This will lead to an imbalance between *LRRK2* and *PINK1* kinase pair, contributing to increased TH and DA levels, elevated generation of toxic DA by-products and DA neuron vulnerability. **c** Vice versa, under *PINK1* mutations, the *PINK1* kinase activity can be impaired, which will also disturb the LRRK2-PINK1 kinase balance, leading to deregulated TH-DA pathway, enhanced generation of toxic DA by-products, and DA neuron vulnerability

Previous studies demonstrated that *LRRK2* and *PINK1* mutations promote dopaminergic neuronal toxicity. The *LRRK2* mutations impair synaptic vesicle endocytosis, leading to alterations of DA metabolism and DA-mediated toxic effects in dopaminergic neurons derived from iPSCs generated from reprogrammed PD patient fibroblasts carrying *LRRK2* mutations [132]. The PINK1 protein is mostly localized in mitochondria; however, extra-mitochondrial fragment of PINK1 can modulate TH expression and DA level in dopaminergic neurons in a PINK1 kinase activity-dependent manner [42]. The overexpression of WT PINK1 has been shown to down-regulate TH expression and DA level to protect human dopaminergic neurons [42]. However, transfection of PD-related PINK1 mutants up-regulated TH and DA levels in dopaminergic neurons, making them vulnerable to oxidative stress [42]. Furthermore, recent findings highlight the vital role of the LRRK2–PINK1 kinase pair in the modulation of the TH–DA pathway in PD pathogenesis [44]. LRRK2 and PINK1 form a functional protein kinase pair to modulate TH and DA levels in dopaminergic neurons, and this observation has been validated in multiple in vitro and in vivo PD models, including human dopaminergic neurons and midbrain organoid models derived from patient cell-induced iPSCs [44]. LRRK2 promotes TH expression and increases DA generation which can be aggravated by *LRRK2* mutations, while WT PINK1 suppresses TH expression and DA generation, which can be abrogated by PD-linked *PINK1* mutations [44]. Furthermore, LRRK2 and PINK1 can facilitate proteasome degradation of each other to reciprocally down-regulate their protein levels, reaching a functional balance [44]. Under physiological conditions, LRRK2 and PINK1 form a functional balance to maintain normal TH expression and DA synthesis in dopaminergic neurons [44]. However, in the presence of *LRRK2* mutations, the LRRK2 kinase activity is increased, leading to up-regulated TH expression and increased DA synthesis [44]. The increased LRRK2 kinase activity will also facilitate UPS degradation of PINK1, impairing functions of PINK1 [44]. This can contribute to the imbalance between the LRRK2–PINK1 kinase pair, leading to up-regulation of TH expression, increased DA synthesis, enhanced DA oxidation and aggravation of DA-specific stress in dopaminergic neurons, and dopaminergic neuron vulnerability [44]. Similarly, in the presence of *PINK1* mutations, the PINK1 kinase activity will be impaired, which also causes the imbalance of the LRRK2–PINK1 kinase pair, leading to disrupted TH–DA pathway and dopaminergic neuron vulnerability [44]. These findings indicate that the LRRK2–PINK1 kinase pair and the TH–DA pathway may be potential therapeutic targets for PD. The impact of the LRRK2–PINK1 kinase pair on the TH–DA pathway, relevant to PD pathogenesis and therapy, is illustrated in Fig. 3.

### *Parkin*

Parkin is a cytosolic protein that functions as a ubiquitin E3 ligase to ubiquitinate target proteins and modulate many cellular processes to protect dopaminergic neurons [133]. Impairment of the E3 ligase activity of Parkin is considered to play a pathogenic role in both sporadic and familial forms of PD [133]. Pathogenic *Parkin* gene mutations can cause selective DA neurodegeneration and early-onset parkinsonism [133]. It is reported that DAQs can covalently modify Parkin protein in dopaminergic neurons, leading to Parkin protein insolubility and inactivation of its E3 ubiquitin ligase activity [82, 134]. DAQs could conjugate with and modify Cys95 and Cys253 residues of Parkin protein, leading to its insolubility, as revealed in post-mortem patient brain samples and in PD models in vivo [82, 134]. Furthermore, the decreased solubility of Parkin protein is suggested to impair autophagy and contribute to the accumulation of α-syn protein [135]. These findings demonstrate the complicated pathological networks among DA toxicity and PD genes in PD pathogenesis.

### *DJ-1*

DJ-1 is a small and highly conserved homodimeric protein of 189 amino acids, commonly expressed in both brain and peripheral tissues [18]. Mutations of *DJ-1* can cause inherent autosomal recessive early-onset PD [18]. It was reported that overexpression of WT DJ-1 enhances cell resistance to DA toxicity and inhibits ROS generation [136]. A recent study indicated that WT DJ-1, rather than its pathogenic L166P mutant protein, protected dopaminergic neurons via inhibiting microglial activation [137]. Glial cells, especially microglia and astrocytes, express DA receptors that can bind DA upon its release into the synaptic cleft by dopaminergic neurons [138–140]. DA activates the DA receptors on glial cells upon binding, leading to ROS generation and release of pro-inflammatory cytokines from glial cells, triggering neuronal damage [138–140]. It was found that the WT DJ-1 tightly interacts with the p65 subunit of nuclear factor-κB (NF-κB) in the cytoplasm to inhibit glial activation and neuroinflammation-mediated neurotoxicity [137]. However, loss of DJ-1 promotes the dissociation between p65 and NF-κB inhibitor α (IκBα), facilitating p65 nuclear translocation, resulting in more microglial activation and aggregation of microglia-mediated neurotoxicity in an NF-κB-dependent manner [137].

However, DA-derived DAQs can covalently modify cysteine residues of DJ-1 protein to inactivate DJ-1 protein [83]. DAQs can react with three cysteine residues of DJ-1 with different profiles. Cys46 residue of DJ-1 protein is not reactive to DAQs. Cys53 is the most reactive residue towards DAQs, forming a covalent dimer without disturbance of the structure. However, modification of Cys106 by DAQs has the most severe effects on DJ-1 protein structure and function, leading to DJ-1 aggregation [83]. Cys106 of DJ-1 protein plays key roles in cellular oxidative stress response and mitochondrial function modulations [141]. Furthermore, the aggregation of DJ-1 protein is implicated in PD pathogenesis and increased insolubility of DJ-1 protein has been identified in sporadic PD patient brains [142].

### *GBA1*

Apart from the abovementioned gene mutations, mutations of the *GBA1* gene, which encodes GCase, a lysosomal enzyme that hydrolyzes glucosylceramide to glucose and ceramide, are among the most common genetic risk factors for the development of PD [17]. Studies have shown a higher frequency of *GBA1* mutations in PD than those of other PD-associated genes, including *LRKK2*, *SNCA* and *PINK1* [143, 144]. GCase protein level and activity are reduced under *GBA1* mutations [145, 146]. Evidence shows that DAQs can directly conjugate with GCase and inhibit its enzymatic activity, leading to lysosomal dysfunction and α-syn protein accumulation [147]. Considering the species-specific differences in DA metabolism, Burbulla et al. increased DA synthesis in mouse midbrain neurons, and recapitulated the pathogenic changes found in human dopaminergic neurons, i.e., mitochondrial oxidant stress which leads to DA oxidation, reduced GCase activity and accumulation of α-syn protein [10, 146, 148]. This highlights a role of DA oxidation in mitochondrial and lysosomal dysfunction in PD pathogenesis.

## Therapeutic strategies against DA toxicity

Despite advances in PD treatment over the past decades (medications, deep brain stimulation approaches, etc.), currently no drug can reverse the progressive loss of dopaminergic neurons in PD patient brains [149]. DA exerts neuron toxicity via its deleterious metabolites, including ROS, DAQs and DOPAL. Both DAQs and DOPAL can react with sulfhydryl groups, and agents containing sulfhydryl groups, namely, GSH, *N*-acetylcysteine (NAC) and *L*-cysteine, are able to competitively conjugate with and detoxify the toxic by-products of DA [5, 150–152]. GSH is an important endogenous ROS-scavenger and DAQ-detoxifier [5, 6, 153]. GSH can inhibit and abrogate DA auto-oxidation and enzyme-catalyzed oxidation to suppress the production of reactive ROS and DAQs [5, 6, 153]. GSH can conjugate with DAQs to form GSH-DAQ conjugates, evidenced by the detection of various GSH-DAQ conjugates in post-mortem PD brains [5, 150, 154–156]. Meanwhile, reduced levels of GSH were identified in post-mortem PD brains, suggesting impaired GSH defense in PD [157]. Moreover, DAQs can be detoxified through reactions with NAC or* L*-cysteine to form DAQ conjugation adducts [151, 152]. NAC, an antioxidant and a precursor for GSH, has been used in clinic to improve motor and mental abilities of PD patients, possibly by restoring dopaminergic neuron functions [158, 159]. Recent findings have shown that DAQs can also react and conjugate with hydroxyl groups, especially those within phenol rings [26]. Our recent findings indicate that tea polyphenols can protect dopaminergic neurons via suppression of DA oxidation, reaction with DAQs, inhibition of MAOB, and modulations of the anti-oxidative signaling pathways [26]. The protective potency of tea polyphenols is positively correlated with the number of phenolic hydroxyl groups in their phenol rings [26]. Tea polyphenols with more ring structures and hydroxyl groups are more potent to protect against DA-induced toxicity [26]. Furthermore, the protection of tea polyphenols is more potent than that of sulfhydryl group-containing compounds, including GSH [26]. These findings are promising. In the future, more potent DAQ-detoxification compounds with blood–brain barrier penetrating abilities and versatile protective functions may be identified and developed for PD therapeutic use.

DOPAL produced from DA via MAO catalysis is reactive and toxic to dopaminergic neurons. DOPAL can covalently conjugate to lysine and cysteine residues to induce toxic effects [64, 160]. Lysine mimics have been used in the design of small-molecule inhibitors of histone lysine methyltransferases [161]. Many lysine derivatives have been synthesized and applied in biological research [162, 163]. Therefore, the strategy of scavenging reactive DOPAL aldehydes by an excess of amino molecules or lysine derivatives may protect functional protein lysine residues. As DAQs can also react and conjugate with cysteine and tyrosine residues via MA reaction to induce toxicity, the strategy of protecting protein lysines may be combined with cysteine- and tyrosine-protecting agents, such as GSH, NAC and *L*-cysteine, to achieve optimized therapeutic effects. Future investigations are needed to test and validate these hypotheses.

In a recent study, inhibition of TH by low-dose α-MT initiated at the early stage was able to prevent *LRRK2* G2019S mutation-induced DA neurodegeneration in in vivo PD models [44, 164]. α-MT has been reported to alleviate the degeneration of dopaminergic neurons induced by mutant α-syn and PINK1 [42, 120]. α-MT is a competitive TH inhibitor and has been used clinically for conditions such as hypertension-linked phaeochromocytoma, dystonia, and Huntington's disease [164–166]. Low-dose α-MT is safe with no significant side effects even after prolonged usage (3 years) [166]. Considering its low toxicity and high tolerance among human subjects, low-dosage α-MT seems to be promising in protecting dopaminergic neurons and preventing degeneration of dopaminergic neurons in PD. It will be interesting to determine if α-MT therapy can prevent PD onset in prodromal subjects carrying pathogenic PD gene mutations. More clinical studies are warranted to investigate the therapeutic effects of α-MT and other TH inhibitors in PD.

## Conclusions

Accumulating evidence suggests that DA exerts neurotoxicity via its metabolites including DAQs, DOPAL and ROS. DA oxidation generates deleterious and highly reactive DAQs, which can covalently conjugate with sulfhydryl and hydroxyl groups of protein cysteine and tyrosine residues, respectively, to form MA adductive products. DOPAL can covalently conjugate with lysine residues to form SB adducts. The formation of DAQ-protein and DOPAL-protein conjugates can lead to inactivation of functional proteins, protein misfolding, and even formation of deleterious protein aggregates, which are implicated in PD pathogenesis.

The vulnerability of dopaminergic neurons caused by DAQs can be alleviated by DA oxidation inhibitors and DAQ-detoxification agents, such as iron chelators, sulfhydryl- and hydroxyl-containing compounds, whereas the toxicity of DOPAL can be inhibited by MAO inhibitors, ALDH activators and amino-molecules or lysine derivatives. Therapeutic strategies targeting DA synthesis, transportation, storage and metabolisms, such as inhibition of TH, may be promising. Recent findings suggest that DA by-products can conjugate with α-syn, Parkin, DJ-1 and GCase, leading to loss of functions of proteins and formation of protein aggregates. The LRRK2–PINK1 kinase pair plays a vital role in modulation of the TH–DA pathway. However, mutations of *LRRK2* or *PINK1* can disturb the LRRK2–PINK1 kinase balance, leading to increased TH and DA levels and dopaminergic neuron vulnerability. Therefore, LRRK2 kinase inhibitors and PINK1 kinase activators may help maintain the LRRK2–PINK1 balance and promote dopaminergic neuron survival.

DA contributes to neurodegeneration via complicated mechanisms, which may be counteracted by a combination of mechanisms and agents (Fig. 3). Thus, a cocktail of drugs may have better therapeutic effects. For example, cysteine residue-protecting agents such as NAC, may be combined with lysine residue-protecting agents such as lysine derivatives, to achieve improved neuroprotective effects. These protective agents combined with iron chelators and MAOB inhibitors may be able to alleviate the progressive degeneration of dopaminergic neurons in PD.

## Data Availability

Not applicable.

## Abbreviations

α-MT: α-Methyltyrosine
α-syn: α-Synuclein
ALDH: Aldehyde dehydrogenase
ALR/AR: Aldehyde/aldose reductase
AM: Aminochrome
DA: Dopamine
DAQs: DA quinones
DAT: DA transporter
DOQ: DA-*o*-quinone
L-DOPA: Levodopa
DOPAL: 3,4-Dihydroxyphenylacetaldehyde
DPQ: DOPAL quinone
GBA1: Glucocerebrosidase-1
Gly: Glycine
GSH: Glutathione
HSP27: Heat shock protein 27
IκBα: NF-κB inhibitor α
iPSC: Induced pluripotent stem cell
LRRK2: Leucine-rich repeat kinase 2
Lys: Lysine
L-Cys: l-cysteine
MA: Michael-addition
MAO: Monoamine oxidases
MPP^+^: 1-Methyl-4-phenylpyridinium
MPTP: 1-Methy-4-phenyl-1,2,3,6-tetrahydropyridine
MTP: Mitochondrial transition pore
NAC: *N*-acetylcysteine
NF-κB: Nuclear factor-κB
PD: Parkinson’s disease
PINK1: PTEN-induced kinase 1
ROC: Ras-of-complex proteins
ROS: Reactive oxygen species
SB: Schiff-base
Ser: Serine
SN: Substantia nigra
Thr: Threonine
Tyr: Tyrosine
UPS: Ubiquitin–proteasome system
VMAT2: Vesicular monoamine transporter 2

## References

[1] Meara RJ (1994). Review: the pathophysiology of the motor signs in Parkinson's disease. Age Ageing.

[2] Van Laar VS, Berman SB (2009). Mitochondrial dynamics in Parkinson's disease. Exp Neurol.

[3] Latif S, Jahangeer M, Maknoon Razia D, Ashiq M, Ghaffar A, Akram M (2021). Dopamine in Parkinson's disease. Clin Chim Acta.

[4] Jankovic J (2008). Parkinson’s disease: clinical features and diagnosis. J Neurol Neurosurg Psychiatry.

[5] Zhou Z, Thevapriya S, Chao YX, Lim TM, Tan EK (2016). Dopamine (DA) dependent toxicity relevant to DA neuron degeneration in Parkinson’s disease (PD). Austin J Drug Abuse Addict.

[6] Zhou ZD, Lim TM (2009). Roles of glutathione (GSH) in dopamine (DA) oxidation studied by improved tandem HPLC plus ESI-MS. Neurochem Res.

[7] Antkiewicz-Michaluk L (2002). Endogenous risk factors in Parkinson's disease: dopamine and tetrahydroisoquinolines. Pol J Pharmacol.

[8] Larsen KE, Fon EA, Hastings TG, Edwards RH, Sulzer D (2002). Methamphetamine-induced degeneration of dopaminergic neurons involves autophagy and upregulation of dopamine synthesis. J Neurosci.

[9] Masato A, Plotegher N, Boassa D, Bubacco L (2019). Impaired dopamine metabolism in Parkinson’s disease pathogenesis. Mol Neurodegener.

[10] Burbulla LF, Song P, Mazzulli JR, Zampese E, Wong YC, Jeon S (2017). Dopamine oxidation mediates mitochondrial and lysosomal dysfunction in Parkinson's disease. Science.

[11] Zhou Z, Kerk S, Meng LT (2008). Endogenous dopamine (DA) renders dopaminergic cells vulnerable to challenge of proteasome inhibitor MG132. Free Radic Res.

[12] Zhou ZD, Lim TM (2009). Dopamine (DA) induced irreversible proteasome inhibition via DA derived quinones. Free Radic Res.

[13] Sherer TB, Betarbet R, Stout AK, Lund S, Baptista M, Panov AV (2002). An in vitro model of Parkinson's disease: linking mitochondrial impairment to altered alpha-synuclein metabolism and oxidative damage. J Neurosci.

[14] Salazar J, Mena N, Núñez MT (2006). Iron dyshomeostasis in Parkinson's disease. J Neural Transm Suppl.

[15] Srinivasan E, Chandrasekhar G, Chandrasekar P, Anbarasu K, Vickram AS, Karunakaran R (2021). Alpha-synuclein aggregation in Parkinson's disease. Front Med.

[16] Kumar A, Tamjar J, Waddell AD, Woodroof HI, Raimi OG, Shaw AM (2017). Structure of PINK1 and mechanisms of Parkinson's disease-associated mutations. Elife.

[17] Beavan M, Schapira A (2013). Glucocerebrosidase mutations and the pathogenesis of Parkinson disease. Ann Med.

[18] Repici M, Giorgini F (2019). DJ-1 in Parkinson's disease: clinical insights and therapeutic perspectives. J Clin Med.

[19] Taymans JM (2012). The GTPase function of LRRK2. Biochem Soc Trans.

[20] Stokes AH, Hastings TG, Vrana KE (1999). Cytotoxic and genotoxic potential of dopamine. J Neurosci Res.

[21] Napolitano A, Manini P, d'Ischia M (2011). Oxidation chemistry of catecholamines and neuronal degeneration: an update. Curr Med Chem.

[22] German CL, Baladi MG, McFadden LM, Hanson GR, Fleckenstein AE (2015). Regulation of the dopamine and vesicular monoamine transporters: pharmacological targets and implications for disease. Pharmacol Rev.

[23] Baronio D, Chen Y-C, Decker AR, Enckell L, Fernández-López B, Semenova S (2022). Vesicular monoamine transporter 2 (SLC18A2) regulates monoamine turnover and brain development in zebrafish. Acta Physiol.

[24] Antkiewicz-Michaluk L, Ossowska K, Romańska I, Michaluk J, Vetulani J (2008). 3-Methoxytyramine, an extraneuronal dopamine metabolite plays a physiological role in the brain as an inhibitory regulator of catecholaminergic activity. Eur J Pharmacol.

[25] Graham DG (1978). Oxidative pathways for catecholamines in the genesis of neuromelanin and cytotoxic quinones. Mol Pharmacol.

[26] Zhou ZD, Xie SP, Saw WT, Ho PGH, Wang H, Lei Z (2019). The Therapeutic implications of tea polyphenols against dopamine (da) neuron degeneration in Parkinson's disease (PD). Cells.

[27] Bustamante J, Bredeston L, Malanga G, Mordoh J (1993). Role of melanin as a scavenger of active oxygen species. Pigment Cell Res.

[28] Knörle R (2018). Neuromelanin in Parkinson's disease: from fenton reaction to calcium signaling. Neurotox Res.

[29] Li J, Yang J, Zhao P, Li S, Zhang R, Zhang X (2012). Neuromelanin enhances the toxicity of α-synuclein in SK-N-SH cells. J Neural Transm.

[30] Gonzalez-Sepulveda M, Compte J, Cuadros T, Nicolau A, Guillard-Sirieix C, Peñuelas N (2023). In vivo reduction of age-dependent neuromelanin accumulation mitigates features of Parkinson's disease. Brain.

[31] Marchitti SA, Deitrich RA, Vasiliou V (2007). Neurotoxicity and metabolism of the catecholamine-derived 3,4-dihydroxyphenylacetaldehyde and 3,4-dihydroxyphenylglycolaldehyde: the role of aldehyde dehydrogenase. Pharmacol Rev.

[32] Zhang S, Wang R, Wang G (2019). Impact of dopamine oxidation on dopaminergic neurodegeneration. ACS Chem Neurosci.

[33] Inyushin MY, Huertas A, Kucheryavykh YV, Kucheryavykh LY, Tsydzik V, Sanabria P (2012). L-DOPA uptake in astrocytic endfeet enwrapping blood vessels in rat Brain. Parkinsons Dis.

[34] Lotharius J, Falsig J, van Beek J, Payne S, Dringen R, Brundin P (2005). Progressive degeneration of human mesencephalic neuron-derived cells triggered by dopamine-dependent oxidative stress is dependent on the mixed-lineage kinase pathway. J Neurosci.

[35] Bayersdorfer F, Voigt A, Schneuwly S, Botella JA (2010). Dopamine-dependent neurodegeneration in Drosophila models of familial and sporadic Parkinson's disease. Neurobiol Dis.

[36] Filloux F, Townsend JJ (1993). Pre- and postsynaptic neurotoxic effects of dopamine demonstrated by intrastriatal injection. Exp Neurol.

[37] Hastings TG, Lewis DA, Zigmond MJ (1996). Role of oxidation in the neurotoxic effects of intrastriatal dopamine injections. Proc Natl Acad Sci USA.

[38] Rabinovic AD, Lewis DA, Hastings TG (2000). Role of oxidative changes in the degeneration of dopamine terminals after injection of neurotoxic levels of dopamine. Neuroscience.

[39] Díaz-Véliz G, Mora S, Dossi MT, Gómez P, Arriagada C, Montiel J (2002). Behavioral effects of aminochrome and dopachrome injected in the rat substantia nigra. Pharmacol Biochem Behav.

[40] Touchette JC, Breckenridge JM, Wilken GH, Macarthur H (2016). Direct intranigral injection of dopaminochrome causes degeneration of dopamine neurons. Neurosci Lett.

[41] Burke WJ, Li SW, Williams EA, Nonneman R, Zahm DS (2003). 3,4-Dihydroxyphenylacetaldehyde is the toxic dopamine metabolite in vivo: implications for Parkinson’s disease pathogenesis. Brain Res.

[42] Zhou ZD, Refai FS, Xie SP, Ng SH, Chan CHS, Ho PGH (2014). Mutant PINK1 upregulates tyrosine hydroxylase and dopamine levels, leading to vulnerability of dopaminergic neurons. Free Radic Biol Med.

[43] Vecchio LM, Sullivan P, Dunn AR, Bermejo MK, Fu R, Masoud ST (2021). Enhanced tyrosine hydroxylase activity induces oxidative stress, causes accumulation of autotoxic catecholamine metabolites, and augments amphetamine effects in vivo. J Neurochem.

[44] Zhou ZD, Saw WT, Ho PGH, Zhang ZW, Zeng L, Chang YY (2022). The role of tyrosine hydroxylase–dopamine pathway in Parkinson’s disease pathogenesis. Cell Mol Life Sci.

[45] Caudle WM, Richardson JR, Wang MZ, Taylor TN, Guillot TS, McCormack AL (2007). Reduced vesicular storage of dopamine causes progressive nigrostriatal neurodegeneration. J Neurosci.

[46] Fumagalli F, Gainetdinov RR, Wang YM, Valenzano KJ, Miller GW, Caron MG (1999). Increased methamphetamine neurotoxicity in heterozygous vesicular monoamine transporter 2 knock-out mice. J Neurosci.

[47] Guillot TS, Shepherd KR, Richardson JR, Wang MZ, Li Y, Emson PC (2008). Reduced vesicular storage of dopamine exacerbates methamphetamine-induced neurodegeneration and astrogliosis. J Neurochem.

[48] Sulzer D, Chen TK, Lau YY, Kristensen H, Rayport S, Ewing A (1995). Amphetamine redistributes dopamine from synaptic vesicles to the cytosol and promotes reverse transport. J Neurosci.

[49] LaVoie MJ, Hastings TG (1999). Dopamine quinone formation and protein modification associated with the striatal neurotoxicity of methamphetamine: evidence against a role for extracellular dopamine. J Neurosci.

[50] Lohr KM, Stout KA, Dunn AR, Wang M, Salahpour A, Guillot TS (2015). Increased vesicular monoamine transporter 2 (VMAT2; Slc18a2) protects against methamphetamine toxicity. ACS Chem Neurosci.

[51] Masoud ST, Vecchio LM, Bergeron Y, Hossain MM, Nguyen LT, Bermejo MK (2015). Increased expression of the dopamine transporter leads to loss of dopamine neurons, oxidative stress and l-DOPA reversible motor deficits. Neurobiol Dis.

[52] Chen L, Ding Y, Cagniard B, Van Laar AD, Mortimer A, Chi W (2008). Unregulated cytosolic dopamine causes neurodegeneration associated with oxidative stress in mice. J Neurosci.

[53] Wey MC, Fernandez E, Martinez PA, Sullivan P, Goldstein DS, Strong R (2012). Neurodegeneration and motor dysfunction in mice lacking cytosolic and mitochondrial aldehyde dehydrogenases: implications for Parkinson's disease. PLoS ONE.

[54] Fitzmaurice AG, Rhodes SL, Lulla A, Murphy NP, Lam HA, O’Donnell KC (2013). Aldehyde dehydrogenase inhibition as a pathogenic mechanism in Parkinson disease. Proc Natl Acad Sci USA.

[55] Danielson SR, Andersen JK (2008). Oxidative and nitrative protein modifications in Parkinson's disease. Free Radic Biol Med.

[56] Asanuma M, Miyazaki I, Ogawa N (2003). Dopamine- or L-DOPA-induced neurotoxicity: the role of dopamine quinone formation and tyrosinase in a model of Parkinson's disease. Neurotox Res.

[57] Van Laar VS, Mishizen AJ, Cascio M, Hastings TG (2009). Proteomic identification of dopamine-conjugated proteins from isolated rat brain mitochondria and SH-SY5Y cells. Neurobiol Dis.

[58] Wang N, Wang Y, Yu G, Yuan C, Ma J (2011). Quinoprotein adducts accumulate in the substantia Nigra of aged rats and correlate with dopamine-induced toxicity in SH-SY5Y cells. Neurochem Res.

[59] Hayashi J, Ton J, Negi S, Stephens D, Pountney DL, Preiss T (2021). The effect of oxidized dopamine on the structure and molecular chaperone function of the small heat-shock proteins, αB-crystallin and Hsp27. Int J Mol Sci.

[60] Miyazaki I, Asanuma M (2009). Approaches to prevent dopamine quinone-induced neurotoxicity. Neurochem Res.

[61] Jana S, Sinha M, Chanda D, Roy T, Banerjee K, Munshi S (2011). Mitochondrial dysfunction mediated by quinone oxidation products of dopamine: Implications in dopamine cytotoxicity and pathogenesis of Parkinson's disease. Biochim Biophys Acta.

[62] Muñoz P, Huenchuguala S, Paris I, Segura-Aguilar J (2012). Dopamine oxidation and autophagy. Parkinsons Dis.

[63] Biosa A, Arduini I, Soriano ME, Giorgio V, Bernardi P, Bisaglia M (2018). Dopamine oxidation products as mitochondrial endotoxins, a potential molecular mechanism for preferential neurodegeneration in Parkinson’s disease. ACS Chem Neurosci.

[64] Rees JN, Florang VR, Eckert LL, Doorn JA (2009). Protein reactivity of 3,4-dihydroxyphenylacetaldehyde, a toxic dopamine metabolite, is dependent on both the aldehyde and the catechol. Chem Res Toxicol.

[65] Anderson DG, Mariappan SVS, Buettner GR, Doorn JA (2011). Oxidation of 3,4-dihydroxyphenylacetaldehyde, a toxic dopaminergic metabolite, to a semiquinone radical and an ortho-quinone. J Biol Chem.

[66] Panneton WM, Kumar VB, Gan Q, Burke WJ, Galvin JE (2010). The neurotoxicity of DOPAL: behavioral and stereological evidence for its role in Parkinson disease pathogenesis. PLoS ONE.

[67] Rees JN, Florang VR, Anderson DG, Doorn JA (2007). Lipid peroxidation products inhibit dopamine catabolism yielding aberrant levels of a reactive intermediate. Chem Res Toxicol.

[68] Follmer C, Coelho-Cerqueira E, Yatabe-Franco DY, Araujo GD, Pinheiro AS, Domont GB (2015). Oligomerization and membrane-binding properties of covalent adducts formed by the interaction of α-synuclein with the toxic dopamine metabolite 3,4-dihydroxyphenylacetaldehyde (DOPAL). J Biol Chem.

[69] Masato A, Plotegher N, Terrin F, Sandre M, Faustini G, Thor A (2023). DOPAL initiates αSynuclein-dependent impaired proteostasis and degeneration of neuronal projections in Parkinson’s disease. NPJ Parkinson's Dis.

[70] Plotegher N, Bubacco L (2016). Lysines, Achilles' heel in alpha-synuclein conversion to a deadly neuronal endotoxin. Ageing Res Rev.

[71] Yunden J, Yehonatan S, Patti S, Risa I, David SG (2018). 3,4-Dihydroxyphenylacetaldehyde-induced protein modifications and their mitigation by N-acetylcysteine. J Pharmacol Exp Ther.

[72] Alqaraghuli HGJ, Kashanian S, Rafipour R, Mansouri K (2019). Dopamine-conjugated apoferritin protein nanocage for the dual-targeting delivery of epirubicin. Nanomed J.

[73] Plotegher N, Berti G, Ferrari E, Tessari I, Zanetti M, Lunelli L (2017). DOPAL derived alpha-synuclein oligomers impair synaptic vesicles physiological function. Sci Rep.

[74] Werner-Allen JW, Monti S, DuMond JF, Levine RL, Bax AJB (2018). Isoindole linkages provide a pathway for DOPAL-mediated cross-linking of α-synuclein. Biochemistry.

[75] Werner-Allen JW, DuMond JF, Levine RL, Bax A (2016). Toxic dopamine metabolite DOPAL forms an unexpected dicatechol pyrrole adduct with lysines of α-synuclein. Angew Chem Int Ed Engl.

[76] Martinez-Vicente M, Talloczy Z, Kaushik S, Massey AC, Mazzulli J, Mosharov EV (2008). Dopamine-modified alpha-synuclein blocks chaperone-mediated autophagy. J Clin Investig.

[77] Cappai R, Leck S-L, Tew DJ, Williamson NA, Smith DP, Galatis D (2005). Dopamine promotes α-synuclein aggregation into SDS-resistant soluble oligomers via a distinct folding pathway. FASEB J.

[78] Jinsmaa Y, Isonaka R, Sharabi Y, Goldstein DS (2020). 3,4-Dihydroxyphenylacetaldehyde Is more efficient than dopamine in oligomerizing and quinonizing α-synuclein. J Pharmacol Exp Ther.

[79] Bisaglia M, Mammi S, Bubacco L (2007). Kinetic and structural analysis of the early oxidation products of dopamine: analysis of the interactions with alpha-synuclein. J Biol Chem.

[80] Bisaglia M, Tosatto L, Munari F, Tessari I, de Laureto PP, Mammi S (2010). Dopamine quinones interact with α-synuclein to form unstructured adducts. Biochem Biophys Res Commun.

[81] Smith L, Mullin S, Schapira AHV (2017). Insights into the structural biology of Gaucher disease. Exp Neurol.

[82] LaVoie MJ, Ostaszewski BL, Weihofen A, Schlossmacher MG, Selkoe DJ (2005). Dopamine covalently modifies and functionally inactivates parkin. Nat Med.

[83] Girotto S, Sturlese M, Bellanda M, Tessari I, Cappellini R, Bisaglia M (2012). Dopamine-derived quinones affect the structure of the redox sensor DJ-1 through modifications at Cys-106 and Cys-53. J Biol Chem.

[84] Whitehead RE, Ferrer JV, Javitch JA, Justice JB (2001). Reaction of oxidized dopamine with endogenous cysteine residues in the human dopamine transporter. J Neurochem.

[85] Hauser DN, Dukes AA, Mortimer AD, Hastings TG (2013). Dopamine quinone modifies and decreases the abundance of the mitochondrial selenoprotein glutathione peroxidase 4. Free Radic Biol Med.

[86] Paris I, Perez-Pastene C, Cardenas S, Iturriaga-Vasquez P, Muñoz P, Couve E (2010). Aminochrome induces disruption of actin, alpha-, and beta-tubulin cytoskeleton networks in substantia-nigra-derived cell line. Neurotox Res.

[87] Wakamatsu K, Nakao K, Tanaka H, Kitahori Y, Tanaka Y, Ojika M (2019). The Oxidative pathway to dopamine-protein conjugates and their pro-oxidant activities: implications for the neurodegeneration of parkinson’s disease. Int J Mol Sci.

[88] Ferrari E, Engelen M, Monzani E, Sturini M, Girotto S, Bubacco L (2013). Synthesis and structural characterization of soluble neuromelanin analogs provides important clues to its biosynthesis. J Biol Inorg Chem.

[89] Vanle BC, Florang VR, Murry DJ, Aguirre AL, Doorn JA (2017). Inactivation of glyceraldehyde-3-phosphate dehydrogenase by the dopamine metabolite, 3,4-dihydroxyphenylacetaldehyde. Biochem Biophys Res Commun.

[90] Nicolis S, Zucchelli M, Monzani E, Casella L (2008). Myoglobin modification by enzyme-generated dopamine reactive species. Chemistry.

[91] Yu G, Liu H, Zhou W, Zhu X, Yu C, Wang N (2015). In vivo protein targets for increased quinoprotein adduct formation in aged substantia nigra. Exp Neurol.

[92] Belluzzi E, Bisaglia M, Lazzarini E, Tabares LC, Beltramini M, Bubacco L (2012). Human SOD2 modification by dopamine quinones affects enzymatic activity by promoting its aggregation: possible implications for Parkinson’s disease. PLoS ONE.

[93] Kuhn DM, Arthur RE, Thomas DM, Elferink LA (1999). Tyrosine hydroxylase is inactivated by catechol-quinones and converted to a redox-cycling quinoprotein. J Neurochem.

[94] Xu Y, Stokes AH, Roskoski R, Vrana KE (1998). Dopamine, in the presence of tyrosinase, covalently modifies and inactivates tyrosine hydroxylase. J Neurosci Res.

[95] Mexas LM, Florang VR, Doorn JA (2011). Inhibition and covalent modification of tyrosine hydroxylase by 3,4-dihydroxyphenylacetaldehyde, a toxic dopamine metabolite. Neurotoxicology.

[96] Borsche M, Pereira SL, Klein C, Grünewald A (2021). Mitochondria and Parkinson's disease: clinical, molecular, and translational aspects. J Parkinsons Dis.

[97] Li JL, Lin TY, Chen PL, Guo TN, Huang SY, Chen CH (2021). Mitochondrial function and Parkinson's disease: from the perspective of the electron transport chain. Front Mol Neurosci.

[98] Hastings TG (2009). The role of dopamine oxidation in mitochondrial dysfunction: implications for Parkinson's disease. J Bioenerg Biomembr.

[99] O'Callaghan JP, Seidler FJ (1992). 1-Methyl-4-phenyl-1,2,3,6-tetrahydropyridine (MPTP)-induced astrogliosis does not require activation of ornithine decarboxylase. Neurosci Lett.

[100] Zawada WM, Banninger GP, Thornton J, Marriott B, Cantu D, Rachubinski AL (2011). Generation of reactive oxygen species in 1-methyl-4-phenylpyridinium (MPP+) treated dopaminergic neurons occurs as an NADPH oxidase-dependent two-wave cascade. J Neuroinflamm.

[101] Hsieh YC, Mounsey RB, Teismann P (2011). MPP(+)-induced toxicity in the presence of dopamine is mediated by COX-2 through oxidative stress. Naunyn Schmiedebergs Arch Pharmacol.

[102] Gao L, Zhou W, Symmes B, Freed CR (2016). Re-cloning the N27 dopamine cell line to improve a cell culture model of parkinson's disease. PLoS ONE.

[103] Lotharius J, O'Malley KL (2000). The Parkinsonism-inducing drug 1-methyl-4-phenylpyridinium triggers intracellular dopamine oxidation: a novel mehanism of toxicity. J Biol Chem.

[104] Bezard E, Gross CE, Fournier M-C, Dovero S, Bloch B, Jaber M (1999). Absence of MPTP-induced neuronal death in mice lacking the dopamine transporter. Exp Neurol.

[105] Michaeli S, Oz G, Sorce DJ, Garwood M, Ugurbil K, Majestic S (2007). Assessment of brain iron and neuronal integrity in patients with Parkinson's disease using novel MRI contrasts. Mov Disord.

[106] Griffiths PD, Crossman AR (1993). Distribution of iron in the basal ganglia and neocortex in postmortem tissue in Parkinson's disease and Alzheimer's disease. Dementia.

[107] Zhou ZD, Lan YH, Tan EK, Lim TM (2010). Iron species-mediated dopamine oxidation, proteasome inhibition, and dopaminergic cell demise: implications for iron-related dopaminergic neuron degeneration. Free Radic Biol Med.

[108] Dichtl S, Haschka D, Nairz M, Seifert M, Volani C, Lutz O (2018). Dopamine promotes cellular iron accumulation and oxidative stress responses in macrophages. Biochem Pharmacol.

[109] Haavik J, Le Bourdelles B, Martinez A, Flatmark T, Mallet J (1991). Recombinant human tyrosine hydroxylase isozymes. Eur J Biochem.

[110] Xiao G, Zhao M, Liu Z, Du F, Zhou B (2021). Zinc antagonizes iron-regulation of tyrosine hydroxylase activity and dopamine production in Drosophila melanogaster. BMC Biol.

[111] Colamartino M, Padua L, Meneghini C, Leone S, Cornetta T, Testa A (2012). Protective effects of L-dopa and carbidopa combined treatments on human catecholaminergic cells. DNA Cell Biol.

[112] Han SK, Mytilineou C, Cohen G (1996). L-DOPA up-regulates glutathione and protects mesencephalic cultures against oxidative stress. J Neurochem.

[113] Maharaj H, Sukhdev Maharaj D, Scheepers M, Mokokong R, Daya S (2005). l-DOPA administration enhances 6-hydroxydopamine generation. Brain Res.

[114] Melamed E, Offen D, Shirvan A, Djaldetti R, Barzilai A, Ziv I (1998). Levodopa toxicity and apoptosis. Ann Neurol.

[115] Muddapu VR-J, Vijayakumar K, Ramakrishnan K, Chakravarthy VS (2022). A multi-scale computational model of levodopa-induced toxicity in Parkinson's disease. Front Neurosci.

[116] Hörmann P, Delcambre S, Hanke J, Geffers R, Leist M, Hiller K (2021). Impairment of neuronal mitochondrial function by l-DOPA in the absence of oxygen-dependent auto-oxidation and oxidative cell damage. Cell Death Discov.

[117] Chan SW, Dunlop RA, Rowe A, Double KL, Rodgers KJ (2012). L-DOPA is incorporated into brain proteins of patients treated for Parkinson's disease, inducing toxicity in human neuroblastoma cells in vitro. Exp Neurol.

[118] Irwin DJ, Lee VM, Trojanowski JQ (2013). Parkinson's disease dementia: convergence of α-synuclein, tau and amyloid-β pathologies. Nat Rev Neurosci.

[119] Wakabayashi K, Tanji K, Mori F, Takahashi H (2007). The Lewy body in Parkinson's disease: molecules implicated in the formation and degradation of alpha-synuclein aggregates. Neuropathology.

[120] Zhou ZD, Yap BP, Gung AY, Leong SM, Ang ST, Lim TM (2006). Dopamine-related and caspase-independent apoptosis in dopaminergic neurons induced by overexpression of human wild type or mutant alpha-synuclein. Exp Cell Res.

[121] Bisaglia M, Greggio E, Maric D, Miller DW, Cookson MR, Bubacco L (2010). α-Synuclein overexpression increases dopamine toxicity in BE(2)-M17 cells. BMC Neurosci.

[122] Mor DE, Daniels MJ, Ischiropoulos H (2019). The usual suspects, dopamine and alpha-synuclein, conspire to cause neurodegeneration. Mov Disord.

[123] Conway KA, Rochet JC, Bieganski RM, Lansbury PT (2001). Kinetic stabilization of the alpha-synuclein protofibril by a dopamine-alpha-synuclein adduct. Science.

[124] Mor DE, Tsika E, Mazzulli JR, Gould NS, Kim H, Daniels MJ (2017). Dopamine induces soluble α-synuclein oligomers and nigrostriatal degeneration. Nat Neurosci.

[125] Rochet JC, Outeiro TF, Conway KA, Ding TT, Volles MJ, Lashuel HA (2004). Interactions among alpha-synuclein, dopamine, and biomembranes: some clues for understanding neurodegeneration in Parkinson's disease. J Mol Neurosci.

[126] Bisaglia M, Tosatto L, Munari F, Tessari I, de Laureto PP, Mammi S (2010). Dopamine quinones interact with alpha-synuclein to form unstructured adducts. Biochem Biophys Res Commun.

[127] Cookson MR (2010). The role of leucine-rich repeat kinase 2 (LRRK2) in Parkinson's disease. Nat Rev Neurosci.

[128] Wang Y, Zhang X, Chen F, Chen L, Wang J, Xie J (2021). LRRK2-NFATc2 pathway associated with neuroinflammation may be a potential therapeutic target for Parkinson's disease. J Inflamm Res.

[129] Seol W, Nam D, Son I (2019). Rab GTPases as physiological substrates of LRRK2 kinase. Exp Neurobiol.

[130] Simpson C, Vinikoor-Imler L, Nassan FL, Shirvan J, Lally C, Dam T (2022). Prevalence of ten LRRK2 variants in Parkinson's disease: a comprehensive review. Parkinsonism Relat Disord.

[131] Rakovic A, Grünewald A, Seibler P, Ramirez A, Kock N, Orolicki S (2010). Effect of endogenous mutant and wild-type PINK1 on Parkin in fibroblasts from Parkinson disease patients. Hum Mol Genet.

[132] Nguyen M, Krainc D (2018). LRRK2 phosphorylation of auxilin mediates synaptic defects in dopaminergic neurons from patients with Parkinson’s disease. Proc Natl Acad Sci USA.

[133] Dawson TM, Dawson VL (2010). The role of parkin in familial and sporadic Parkinson's disease. Mov Disord.

[134] Tokarew JM, El-Kodsi DN, Lengacher NA, Fehr TK, Nguyen AP, Shutinoski B (2021). Age-associated insolubility of parkin in human midbrain is linked to redox balance and sequestration of reactive dopamine metabolites. Acta Neuropathol.

[135] Lonskaya I, Hebron ML, Algarzae NK, Desforges N, Moussa CE (2013). Decreased parkin solubility is associated with impairment of autophagy in the nigrostriatum of sporadic Parkinson's disease. Neuroscience.

[136] Lev N, Ickowicz D, Barhum Y, Lev S, Melamed E, Offen D (2009). DJ-1 protects against dopamine toxicity. J Neural Transm.

[137] Lin Z, Chen C, Yang D, Ding J, Wang G, Ren H (2021). DJ-1 inhibits microglial activation and protects dopaminergic neurons in vitro and in vivo through interacting with microglial p65. Cell Death Dis.

[138] Xia Q-P, Cheng Z-Y, He L (2019). The modulatory role of dopamine receptors in brain neuroinflammation. Int Immunopharmacol.

[139] Dominguez-Meijide A, Rodriguez-Perez AI, Diaz-Ruiz C, Guerra MJ, Labandeira-Garcia JL (2017). Dopamine modulates astroglial and microglial activity via glial renin-angiotensin system in cultures. Brain Behav Immun.

[140] Mastroeni D, Grover A, Leonard B, Joyce JN, Coleman PD, Kozik B (2009). Microglial responses to dopamine in a cell culture model of Parkinson's disease. Neurobiol Aging.

[141] Canet-Avilés RM, Wilson MA, Miller DW, Ahmad R, McLendon C, Bandyopadhyay S (2004). The Parkinson's disease protein DJ-1 is neuroprotective due to cysteine-sulfinic acid-driven mitochondrial localization. Proc Natl Acad Sci USA.

[142] West AB, Moore DJ, Biskup S, Bugayenko A, Smith WW, Ross CA (2005). Parkinson's disease-associated mutations in leucine-rich repeat kinase 2 augment kinase activity. Proc Natl Acad Sci USA.

[143] Sidransky E, Nalls MA, Aasly JO, Aharon-Peretz J, Annesi G, Barbosa ER (2009). Multicenter analysis of glucocerebrosidase mutations in Parkinson's disease. N Engl J Med.

[144] Sidransky E, Lopez G (2012). The link between the GBA gene and parkinsonism. Lancet Neurol.

[145] Burbulla LF, Jeon S, Zheng J, Song P, Silverman RB, Krainc D (2019). A modulator of wild-type glucocerebrosidase improves pathogenic phenotypes in dopaminergic neuronal models of Parkinson's disease. Sci Transl Med.

[146] Mazzulli JR, Zunke F, Tsunemi T, Toker NJ, Jeon S, Burbulla LF (2016). Activation of β-glucocerebrosidase reduces pathological α-synuclein and restores lysosomal function in Parkinson's patient midbrain neurons. J Neurosci.

[147] Monzani E, Nicolis S, Dell'Acqua S, Capucciati A, Bacchella C, Zucca FA (2019). Dopamine, oxidative stress and protein-quinone modifications in Parkinson's and other neurodegenerative diseases. Angew Chem Int Ed Engl.

[148] Chiasserini D, Paciotti S, Eusebi P, Persichetti E, Tasegian A, Kurzawa-Akanbi M (2015). Selective loss of glucocerebrosidase activity in sporadic Parkinson's disease and dementia with Lewy bodies. Mol Neurodegener.

[149] Nemade D, Subramanian T, Shivkumar V (2021). An update on medical and surgical treatments of Parkinson's disease. Aging Dis.

[150] Zhou ZD, Lim TM (2010). Glutathione conjugates with dopamine-derived quinones to form reactive or non-reactive glutathione-conjugates. Neurochem Res.

[151] Banaclocha MM (2001). Therapeutic potential of *N*-acetylcysteine in age-related mitochondrial neurodegenerative diseases. Med Hypotheses.

[152] Sulzer D, Zecca L (2000). Intraneuronal dopamine-quinone synthesis: a review. Neurotox Res.

[153] Jia Z, Zhu H, Misra HP, Li Y (2008). Potent induction of total cellular GSH and NQO1 as well as mitochondrial GSH by 3H–1,2-dithiole-3-thione in SH-SY5Y neuroblastoma cells and primary human neurons: protection against neurocytotoxicity elicited by dopamine, 6-hydroxydopamine, 4-hydroxy-2-nonenal, or hydrogen peroxide. Brain Res.

[154] Spencer JP, Jenner P, Daniel SE, Lees AJ, Marsden DC, Halliwell B (1998). Conjugates of catecholamines with cysteine and GSH in Parkinson's disease: possible mechanisms of formation involving reactive oxygen species. J Neurochem.

[155] Spencer JP, Jenner P, Halliwell B (1995). Superoxide-dependent depletion of reduced glutathione by L-DOPA and dopamine. Relevance to Parkinson's disease. NeuroReport.

[156] Rosengren E, Linder-Eliasson E, Carlsson A (1985). Detection of 5-S-cysteinyldopamine in human brain. J Neural Transm.

[157] Martin HL, Teismann P (2009). Glutathione—a review on its role and significance in Parkinson's disease. FASEB J.

[158] Monti DA, Zabrecky G, Kremens D, Liang TW, Wintering NA, Bazzan AJ (2019). *N*-Acetyl cysteine is associated with dopaminergic improvement in Parkinson's disease. Clin Pharmacol Ther.

[159] Coles LD, Tuite PJ, Öz G, Mishra UR, Kartha RV, Sullivan KM (2018). Repeated-dose oral *N*-acetylcysteine in Parkinson's disease: pharmacokinetics and effect on brain glutathione and oxidative stress. Clin Pharmacol Ther.

[160] Anderson DG, Florang VR, Schamp JH, Buettner GR, Doorn JA (2016). Antioxidant-mediated modulation of protein reactivity for 3,4-dihydroxyphenylacetaldehyde, a toxic dopamine metabolite. Chem Res Toxicol.

[161] Chang Y, Ganesh T, Horton JR, Spannhoff A, Liu J, Sun A (2010). Adding a lysine mimic in the design of potent inhibitors of histone lysine methyltransferases. J Mol Biol.

[162] Flügel V, Vrabel M, Schneider S (2014). Structural basis for the site-specific incorporation of lysine derivatives into proteins. PLoS ONE.

[163] Narang N, Sato T (2021). Liquid–liquid phase separation and self-assembly of a lysine derivative Fmoc-l-lysine in water-DMSO mixtures. Polym J.

[164] Burke RE, Fahn S, Mayeux R, Weinberg H, Louis K, Willner JH (1981). Neuroleptic malignant syndrome caused by dopamine-depleting drugs in a patient with Huntington disease. Neurology.

[165] Brogden RN, Heel RC, Speight TM, Avery GS (1981). alpha-Methyl-*p*-tyrosine: a review of its pharmacology and clinical use. Drugs.

[166] Ankenman R, Salvatore MF (2007). Low dose alpha-methyl-para-tyrosine (AMPT) in the treatment of dystonia and dyskinesia. J Neuropsychiatry Clin Neurosci.

